# Alcohol Marketing, Drunkenness, and Problem Drinking among Zambian Youth: Findings from the 2004 Global School-Based Student Health Survey

**DOI:** 10.1155/2011/497827

**Published:** 2011-03-14

**Authors:** Monica H. Swahn, Bina Ali, Jane B. Palmier, George Sikazwe, John Mayeya

**Affiliations:** ^1^Institute of Public Health, Georgia State University, P.O. Box 3995, Atlanta, GA 30302-3995, USA; ^2^Health Promotion, Ministry of Health, P.O. Box 30205, Lusaka, Zambia; ^3^Department of Health Promotion, University of Zambia, P.O. Box 32379, Lusaka, Zambia

## Abstract

This study examines the associations between alcohol marketing strategies, alcohol education including knowledge about dangers of alcohol and refusal of alcohol, and drinking prevalence, problem drinking, and drunkenness. Analyses are based on the Global School-Based Student Health Survey (GSHS) conducted in Zambia (2004) of students primarily 11 to 16 years of age (N = 2257). Four statistical models were computed to test the associations between alcohol marketing and education and alcohol use, while controlling for possible confounding factors. Alcohol marketing, specifically through providing free alcohol through a company representative, was associated with drunkenness (AOR = 1.49; 95% CI: 1.09–2.02) and problem drinking (AOR = 1.41; 95% CI: 1.06–1.87) among youth after controlling for demographic characteristics, risky behaviors, and alcohol education. However, alcohol education was not associated with drunkenness or problem drinking. These findings underscore the importance of restricting alcohol marketing practices as an important policy strategy for reducing alcohol use and its dire consequences among vulnerable youth.

## 1. Introduction

Alcohol use is a serious risk factor for chronic diseases and injuries worldwide [1]. Globally, alcohol causes 1.8 million or 3.2% of all deaths and accounts for 4.0% of the disease burden [2]. Studies have reported that alcohol use is associated with alcohol dependence [3], other substance use, criminal activity [4], unintentional injuries [5, 6], involvement in physical fights [7], suicidal ideation and attempts [8–10], and risk of human immunodeficiency disease [11, 12]. Indeed, the World Health Organization (WHO) has recently prioritized reducing the harmful use of alcohol globally through monitoring and technical support [13]. Although data is relatively limited, it is clear that the disease burden related to alcohol use is especially great among low-income and middle-income populations and countries where alcohol consumption is increasing and injury rates are high due to limited implementation of public health policies and prevention strategies [1, 2]. For example, in Africa, alcohol use has been found to be associated with road traffic crashes [14], unprotected sex [15–18], and mental disorders [19]. In Zambia specifically, 40.8% of adolescents (36.7% of boys and 45.2% of girls) have ever drunk alcohol [20].

Other studies have shown that alcohol use is affected by individual and environmental factors. Correlates of alcohol use include demographic factors such as gender, age, monthly income, living arrangement, but also attitudes toward alcohol use, perceived susceptibility of alcohol use, perceived self-efficacy, peer drinking, relatives drinking, accessibility of alcohol, and exposure to either antialcohol campaigns or to alcohol advertising as well as ownership of alcohol promotional items [21–25]. A study on alcohol use in Africa identified that alcohol use and risky sexual behaviors are linked to drinking venues and alcohol serving establishments, sexual coercion, and poverty [16]. In Zambia, alcohol use among youth has been found to be significantly associated with suicide ideation and physical fighting [26]. These findings indicate that alcohol use is a pressing public health issue in Africa that is linked to other health-risk behaviors and adverse outcomes.

Alcohol marketing is one of the major risk factors for alcohol use. Exposure to alcohol advertising and ownership of alcohol promotional items, such as t-shirts, lighters, matches, hats, or sunglasses with an alcohol brand name on it, increase the risk of alcohol use among adolescents [22, 27]. Alcohol marketing influences youth's attitudes and perceptions about alcohol, which are related to expectancies and intentions to consume alcohol beverages [28, 29]. In general, liking alcohol advertisements, such as displays of alcohol products in retail stores, retail store discounts, price specials, and coupons, is associated with an increased likelihood to use alcohol [30, 31]. One study found that exposure to alcohol advertising in youth predicts youth's intentions of alcohol consumption up to two years later [32]. Another study found that ownership of alcohol-branded merchandise was associated with a range of high-risk behaviors, poor academic performance, and early alcohol use initiation among youth [23].

The Marin Institute for the Prevention of Alcohol and Other Drug Problems, a group dedicated to respond to the alcohol industry and their marketing practices primarily in the U.S., notes that the alcohol industry spends more than $4.5 billion each year on marketing its products [33]. Alcohol marketing practices that are aimed directly at youth and that are outside of the home (e.g., billboards, advertisements on public transit vehicles, buildings, newspaper stands, and kiosks) are particularly troubling because parents cannot typically shield their children from those exposures. Moreover, spending on out-of-home advertising in the U.S. by major alcohol companies has increased more than $2 billion over the past three years [32]. Research demonstrates that alcohol advertising and marketing of alcohol products clearly increase intent to use as well as actual alcohol use among adolescents [28, 29, 31, 32]. However, there is a dearth of information about alcohol marketing practices and their influence, specifically on youth in Africa. 

The purpose of this study is to examine the prevalence of alcohol marketing and alcohol education exposure in a nationally representative sample of youth in Zambia. Moreover, the study examines if there are significant associations between alcohol marketing and alcohol education and heavy alcohol use including drunkenness and problem drinking among Zambian youth. Findings from this study will be important for prevention efforts that seek to reduce alcohol use among youth [13]. 

## 2. Methods

The current study is based on the Global School-based Student Health Survey, conducted in Zambia in 2004 among students in grades 7–10 (*N* = 2257). The GSHS was developed and supported by the World Health Organization in collaboration with the United Nations Children's Fund, the United Nations Educational, Scientific, and Cultural Organization, the Joint United Nations Programme on HIV/AIDS, and with technical assistance from the Centers for Disease Control and Prevention [34]. The goal of the GSHS is to provide data on health behaviors and relevant risk and protective factors among students across all regions served by the United Nations. Country-specific questionnaires, fact sheets, public-use data files, documentation, and reports are publicly available from the Centers for Disease Control and Prevention and the World Health Organization and have been described elsewhere [20, 35, 36].

Briefly, the GSHS is comprised of a self-administered questionnaire, administered to students about 11–16 years of age. The survey uses a standardized scientific sample selection process, common school-based methodology, and a combination of core questionnaire modules, core-expanded questions, and country-specific questions. The current analyses are based on the restricted data file that includes an expanded list of questions. The school response rate was 93% and student response rate was 75%, yielding an overall response rate of 70%.

### 2.1. Measures

Two outcome measures were included: drunkenness and problem drinking. Drunkenness was assessed through students' reports of the number of times they had gotten drunk during their lifetime on a 4-item scale ranging from 0 times to 10 or more times. Problem drinking was assessed as the number of times they had a hang-over, felt sick, got into trouble with family or friends, missed school, and got into fights due to alcohol use on a 4-item scale ranging from 0 times to 10 or more times. Responses to either outcome measure were dichotomized to reflect none versus any problem drinking behavior.

The alcohol marketing factors included were exposure to alcohol on television, videos, or movies, or exposure to alcohol products via billboard advertisements, and having been provided free alcohol drinks by an alcohol company representative. Responses to these questions were dichotomized to indicate any exposure versus none for each of the three marketing variables. Exposure to alcohol education was measured through questions assessing whether or not they had received alcohol education in school that addressed the dangers of alcohol use and also knowing how to refuse alcohol.

The analyses controlled for the following potential confounders: current alcohol use, bullying victimization, sadness, lack of friends, missing school, lack of parental monitoring, and illicit drug use. Each preceding variable was dichotomized to reflect none versus any involvement or exposure to the particular factor measured.  Table 1 outlines the wording of each measure and its prevalence. 

### 2.2. Analysis

Logistic regression analyses were computed to determine the associations between alcohol marketing, alcohol education, and drunkenness and drinking problem behaviors. For each of the two outcome variables, four models were created and analyzed. Model 1 included demographic characteristics and levels of alcohol use (i.e., sex, age, alcohol use in the past 30 days, and usual amount of alcohol use). Model 2 included variables from Model 1 along with individual psychosocial factors as well as factors reflecting peer and family environment, (i.e., bully victimization, sadness, lack of friends, missing school, lack of parental monitoring, and illicit drug use). Model 3 included variables from Model 1 and Model 2 in addition to factors related to alcohol marketing (i.e., seeing actors drink, alcohol advertisements on billboards, and being offered alcohol from an alcohol company representative). Model 4 included variables from Model 1, Model 2, and Model 3 and protective factors for adverse health outcome (i.e., alcohol education, including knowing the danger of alcohol and refusing alcoholic beverages).

For variables where the amount of missing data exceeded 5 percent, the commonly used missing-indicator method was applied [37, 38]. In this method, a dummy category is created to reflect the missing data and thereby including nearly all participants in the analyses rather than omitting them using the default listwise deletion used in the logistic regression computation. While no statistical findings or associations are reported on the missing data, the Odds Ratio would be interpreted as the risk for the outcome for those with missing data relative to the reference category. Analyses were conducted with the SAS 9.2 and SUDAAN 10 statistical software packages to accommodate the sampling design and produce weighted estimates. 

Institutional Review Board approval was obtained from the Georgia State University to conduct these secondary analyses. Ethical review board approval was originally also obtained from the Ministries of Health and Education in Zambia to conduct the study. Informed consent was collected from students and confidentiality was upheld by allowing for anonymity and voluntary participation as per research ethics requirements. 

## 3. Results

The sample consisted of 52.3% boys and 47.7% girls (*N* = 2257) and represented a weighted count of students in Zambia (*N* = 371,194).  Table 1 shows the prevalence for each variable examined. The bivariate associations between sex, age, alcohol marketing, and alcohol education with current alcohol use, problem drinking, and drunkenness are presented in Table 2. Boys were less likely than girls to report either current alcohol use or drunkenness. There were no sex differences with respect to problem drinking. Overall, age was not an important factor for current alcohol use, drunkenness, or problem drinking. Exposure to alcohol marketing through billboards was associated with fewer reports of current alcohol use and drunkenness. However, being offered free drinks through an alcohol company representative significantly increased reports of current alcohol use (OR = 4.37; 95% CI: 3.21–5.95), drunkenness (OR = 3.02; 95% CI: 2.34–3.90), and problem drinking (OR = 2.51; 95% CI: 1.93–3.27). Having received alcohol education that addressed the dangers of alcohol reduced reports of drunkenness and problem drinking (Table 2).

Multivariate analyses presented in Table 3 show that current alcohol use was the most important correlate of drunkenness across the four models computed. In Models 2 through 4, having missed school and other drug use were also strongly associated with drunkenness. In Model 3, which examined the potential role of alcohol marketing factors, having received free alcohol from a company representative was statistically significantly associated with drunkenness after controlling for demographic characteristics, personal competencies, peer environment, and family environment in Model 3 (AOR = 1.43; 95% CI: 1.07–1.90). Having received free alcohol remained significantly associated with drunkenness even in Model 4 (AOR = 1.49; 95% CI: 1.09–2.02) which also controlled for alcohol education. Alcohol education was not found to be a significant factor related to drunkenness in the multivariate model (Table 3).

The same set of models was also computed for problem drinking (Table 4). Again, current alcohol use was the most important correlate of problem drinking across the four models computed. In Models 2 through 4, bullying victimization, having missed school, and other drug use were also strongly associated with problem drinking. In Model 3, which examined the potential role of alcohol marketing factors, having received free alcohol from a company representative was statistically significantly associated with problem drinking after controlling for demographic characteristics, personal competencies, peer environment, and family environment in Model 3 (AOR: 1.43; 95% CI: 1.07–1.90). Having received free alcohol remained significantly associated with problem drinking even in Model 4 (AOR = 1.41; 95% CI: 1.06–1.87) which also controlled for alcohol education. Alcohol education was not found to be a significant factor related to drinking problem behaviors in the multivariate model (Table 4). 

## 4. Discussion

This study examined the prevalence of exposure to alcohol education and alcohol marketing practices among youth in Zambia. The findings show that many of the youth have received alcohol education; 41% of students said they had been taught about the dangers of alcohol and 45% reported that they knew how to refuse an alcoholic drink. With respect to alcohol marketing exposure, 24% of students reported seeing alcohol through media, 33% reported exposure to alcohol marketing through billboards, and 30% reported that they had been offered a free drink through an alcohol company representative. These findings show that many students are exposed to alcohol, and even offered free alcohol as a marketing strategy, which should be of grave concern given that these students are very young and vulnerable. 

Another goal of the study was to determine the extent to which alcohol education and alcohol marketing strategies were associated with drunkenness as well as problem drinking. The analyses show that current alcohol use, missed school, drug use, and receiving free alcohol from alcohol companies were the most important correlates of drunkenness. Similarly, current alcohol use, bullying victimization, missed school, drug use, and receiving free alcohol from alcohol companies were the most important correlates of problem drinking. Surprisingly, receiving alcohol education that underscores the dangers of alcohol and knowing how to refuse alcohol when given were not statistically associated with either drunkenness or with problem drinking in multivariate analyses. These findings are troubling and indicate that new strategies are needed to prevent and reduce levels of alcohol use and associated adverse outcomes through other strategies than education.

It is clear from previous research that direct marketing of alcohol products increases alcohol use and problems among youth and those findings are corroborated by the findings in the current study. These findings are also supported by research in the US that shows that even distributing alcohol merchandise to youth predicts their alcohol use [39]. Perhaps, even more importantly, alcohol education did not lessen the association between alcohol marketing and drunkenness or problem drinking indicating that these marketing strategies are very robust. These findings may be related to the limited context of alcohol education in school, since parental guidance has been found as a significant direct and indirect factor that lessens the influences of alcohol advertisement and decreases alcohol use [39]. However, this issue needs to be examined in more detail to determine its applicability across cultures and settings. The current analyses also controlled for the impact of parental monitoring which was not associated with drunkenness or problem drinking.

There are several limitations that should be considered when interpreting the findings of this study. First, the study is based on self-reported data of students in Zambia. Accordingly, the findings may not be generalized to other populations or to youth who are no longer in school and may also reflect under or over reporting in disclosure of sensitive information. Second, the study was conducted in 2004 and, therefore, the findings do not reflect any changes in alcohol marketing practices or educational strategies that may have been implemented after the survey was conducted. However, to the best of the authors' knowledge, this is the most recent nationally representative dataset that has assessed student's exposure to alcohol marketing in Zambia. Third, while our findings show statistically significant associations between marketing practices, other correlates, and drunkenness and problem drinking, more specific temporal ordering cannot be determined, nor can causality be inferred. Moreover, some variables lacked operational definition such as “drunkenness” which is an important limitation. Fourth, since several variables had a substantial amount of missing data, the missing-indicator method was applied [37, 38] to include participants with partially missing data in the analyses. This method, while commonly used, may have biased some of the regression coefficients and impacted the findings [40]. Finally, the study did not include specific measures of other marketing strategies and educational experiences or other factors that may influence or confound the associations observed between alcohol marketing, drunkenness, and problem drinking.

The findings of this study show that at least one of the examined alcohol marketing efforts in Zambia, providing free alcohol to youth, appears to be very effective in influencing drinking behaviors and alcohol problems among youth. Some of these alcohol marketing practices, typically aimed directly to children, have been banned in a few other countries such as the U.S., Sweden, France, and Thailand which may inform policy development in countries where such bans do not exist. Concerns have been expressed locally to underscore the ethical violations of alcohol companies that market and target their products directly to children [41] especially since the legal drinking age is 18 in Zambia. The implications of local concerns as well as the empirical findings from the current study clearly indicate that stricter policies to prevent underage alcohol advertisements are needed as are enforcement of existing policies regarding the legal drinking age. The Report on the Seventh meeting of the Regional Advisory Panel on Impacts of Drug Abuse outlines that as a global strategy to reduce the harmful use of alcohol, mandatory and voluntary regulations of marketing of alcohol products need to be considered and included in a comprehensive strategy [13]. These measures need to be urgently considered and applied given the frequency and levels of exposure to alcohol marketing, in particular, the free distribution of alcohol to youth, as observed in the current study. 

The timing is critical for new policy initiatives and prevention strategies aimed to reduce alcohol use [42]. Recent reports by the alcohol industry indicate that they will produce and sell cheaper alcohol to African population in order to increase its consumer market [43]. A recent report in BusinessWeek [43] noted that SABMiller, the world's second largest brewer, is exploring the “relatively untapped African market” to drive future growth, sales, and profits, which is estimated to be worth more than $3 billion. The goal of targeting low-income consumers and creating affordable brews will be achieved through using local ingredients such as cassava and barley, and also utilizing inexpensive individual-sized packaging to make purchasing alcohol more affordable [43]. While the impact of alcohol cost and distribution was not a factor examined in this study, previous research clearly highlights that affordability of alcohol is strongly linked to alcohol use [44], and that these new industry strategies are likely to have a profound and negative impact on alcohol use and alcohol-related adverse outcomes among youth in Africa.

Given these current events, alcohol advertising in Africa is very likely to increase substantially. With the expansion of large alcohol companies into Zambia and other African countries, the issue of alcohol marketing and national alcohol control policies will need to be addressed by the government and public health experts. Policy and intervention suggestions for agencies provided by the World Health Organization to counteract alcohol marketing and reduce harmful effects of alcohol use include regulating alcohol marketing content and the volume of marketing, regulating marketing in media, regulating sponsorship activities of alcohol industry, restricting or banning alcohol promotions targeting young people, regulating alcohol marketing techniques like social media, developing effective surveillance systems to monitor alcohol marketing, and enforcing marketing restrictions [13]. Clearly, increased efforts and resources including more updated information about youth and their levels of exposure to alcohol advertising and levels of consumption will be needed to counteract these marketing influences. Moreover, it will be necessary to broadly promote and disseminate findings that document the extent of exposure to these marketing and distribution strategies and their associated adverse health outcomes among youth to policy makers so that appropriate countermarketing strategies can be supported and implemented. 

## Figures and Tables

**Table 1:** Variable name, description, and prevalence of factors examined in study.

Variablen	Variable description	*N* = 2257 Wtd.%
Current alcohol use	Students who had at least one drink containing alcohol on one or more days during the past 30 days	42.6%
Problem drinking	Students who ever had a hang-over, felt sick, got into trouble with family or friends, missed school, or got into fights, as a result of drinking alcohol	45.1%
Drunkenness	Students who drank so much alcohol that they were really drunk	42.4%
Bullying victimization	Students who were bullied on one or more days in the past 30 days	63.1%
Sadness	Students who felt so sad or hopeless almost every day for two weeks or more in a row that they stopped doing their usual activities during the past 12 months	53.3%
No friends	Students who have no close friends	15.7%
Missed school	Students who missed classes or school without permission on one or more days during the past 30 days	58.5%
No parental monitoring	Students whose parents or guardians really knew what they were doing with their free time in the past 30 days	35.2%
Illicit drug use	Students who used drugs during their life^1^	36.7%
Alcohol marketing		
Actors	Students who watched actors drinking alcohol on television, videos, or movies	24.4%
Billboards	Students who have seen a few or a lot of advertisements for alcohol on billboards in the past 30 days	33.4%
Provided free alcohol	Students who were ever offered a free drink of alcohol by an alcohol company representative	30.0%
Alcohol education		
Danger of alcohol	Students who were taught in classes the dangers of alcohol use	40.9%
Refuse alcohol	Students who were taught in classes to tell someone they did not want to drink alcohol	44.5%

^1^The types of drugs included in the question were “daga” (cannabis).

**Table 2:** Bivariate associations between demographic characteristics, alcohol marketing, and alcohol education and problem drinking and drunkenness.

	Current alcohol use		Problem drinking		Drunkenness	
Demographic characteristics	%	OR (95% CI)	%	OR (95% CI)	%	OR (95% CI)
Sex						
Boys	38.9	**0.76 (0.59–0.98)**	40.8	0. 73 (0.52–1.02)	38.2	**0.70 (0.55–0.89)**
Girls	45.5	1.00	48.8	1.00	46.8	1.00
Age						
≤13	49.9	1.51 (0.86–2.65)	48.3	1.45 (0.93–2.27)	44.3	1.12 (0.72–1.73)
14	41.7	1.08 (0.75–1.58)	53.1	**1.76 (1.21–2.57)**	45.2	1.16 (0.91–1.47)
15	37.8	0.92 (0.63–1.35)	42.4	1.15 (0.87–1.52)	38.7	0.89 (0.67–1.17)
≥16	39.7	1.00	39.1	1.00	41.6	1.00
Alcohol marketing						
Actors	44.2	1.13 (0.85–1.51)	47.2	1.13 (0.90–1.43)	44.8	1.16 (0.89–1.52)
Billboards	34.5	**0.65 (0.46–0.92)**	43.9	1.00 (0.76–1.31)	36.3	**0.74 (0.56–0.98)**
Provided free alcohol	65.3	**4.37 (3.21–5.95)**	58.9	**2.51 (1.93–3.27)**	58.7	**3.02 (2.34–3.90)**
Alcohol education						
Danger of alcohol	35.2	0.68 (0.44–1.07)	37.7	**0.65 (0.50–0.83)**	34.9	**0.64 (0.46–0.89)**
Refuse alcohol	39.7	0.89 (0.65–1.22)	44.1	0.98 (0.77–1.25)	39.3	0.82 (0.63–1.07)

**Table 3:** Multivariate logistic regression analyses of the associations between demographic characteristics, alcohol marketing and alcohol education and drunkenness.

	Four models predicting drunkenness			
	Model 1	Model 2	Model 3	Model 4
	AOR (95% CI)	AOR (95% CI)	AOR (95% CI)	AOR (95% CI)
Boys	**0.68 (0.51–0.91)**	**0.70 (0.51–0.96)**	**0.70 (0.51–0.96)**	**0.71 (0.52–0.97)**
Girls	1.00	1.00	1.00	1.00
Age ≤ 13	0.84 (0.55–1.28)	0.77 (0.48–1.24)	0.76 (0.46–1.26)	0.78 (0.48–1.26)
Age 14	1.10 (0.86–1.42)	0.92 (0.71–1.20)	0.94 (0.71–1.24)	0.92 (0.69–1.22)
Age 15	0.82 (0.58–1.15)	0.76 (0.52–1.10)	0.75 (0.51–1.09)	0.75 (0.52–1.09)
Age ≥ 16	1.00	1.00	1.00	1.00
Alcohol use past 30 days	**33.13 (22.78–48.18)**	**16.68 (10.90–25.53)**	**15.25 (9.75–23.87)**	**15.11 (9.73–23.46)**
Bullying victimization	—	1.38 (0.96–1.98)	1.35 (0.94–1.92)	1.33 (0.94–1.89)
Sadness	—	1.25 (0.99–1.57)	1.20 (0.95–1.51)	1.23 (0.97–1.56)
No friends	—	0.86 (0.60–1.21)	0.85 (0.60–1.20)	0.85 (0.60–1.22)
Missed school	—	**1.53 (1.09–2.16)**	**1.47 (1.04–2.08)**	**1.47 (1.02–2.10)**
No parental monitoring	—	1.33 (0.97–1.83)	1.30 (0.93–1.81)	1.31 (0.94–1.83)
Drug use	—	**3.18 (2.29–4.41)**	**3.07 (2.18–4.33)**	**3.12 (2.18–4.47)**
Alcohol marketing				
Actors	—	—	1.38 (0.97–1.97)	1.37 (0.96–1.95)
Billboards	—	—	0.87 (0.67–1.15)	0.87 (0.67–1.14)
Provided free alcohol	—	—	**1.45 (1.05–2.00)**	**1.49 (1.09–2.02)**
Alcohol education				
Danger of alcohol	—	—	—	0.88 (0.65–1.20)
Refuse alcohol	—	—	—	0.81 (0.59–1.11)

Each model included all listed variables. Reference categories for each variable are not shown but were those not exposed to or who did not report bullying victimization, sadness, having no friends, missed school, no parental monitoring, drug use, alcohol marketing or alcohol education.

**Table 4:** Multivariate logistic regression analyses of the associations between demographic characteristics, alcohol marketing and alcohol education and problem drinking.

	Four models predicting problem drinking			
	Model 1	Model 2	Model 3	Model 4
	AOR (95% CI)	AOR (95% CI)	AOR (95% CI)	AOR (95% CI)
Boys	0.75 (0.52–1.09)	0.76 (0.54–1.08)	0.77 (0.55–1.09)	0.78 (0.55–1.10)
Girls	1.00	1.00	1.00	1.00
Age ≤ 13	1.20 (0.77–1.88)	1.20 (0.75–1.92)	1.22 (0.76–1.96)	1.23 (0.78–1.97)
Age 14	**1.99 (1.31–3.03)**	**1.88 (1.23–2.88)**	**1.92 (1.26–2.93)**	**1.93 (1.26–2.97)**
Age 15	1.20 (0.87–1.66)	1.15 (0.78–1.68)	1.14 (0.77–1.67)	1.14 (0.78–1.67)
Age ≥ 16	1.00	1.00	1.00	1.00
Alcohol use past 30 days	**12.35 (8.67–17.59)**	**5.16 (3.42–7.80)**	**4.87 (3.26–7.29)**	**4.90 (3.26**–**7.37)**
Bullying victimization	—	**2.10 (1.50**–**2.92)**	**2.04 (1.46**–**2.86)**	**2.02 (1.43**–**2.84)**
Sadness	—	1.15 (0.94–1.40)	1.10 (0.90–1.35)	1.12 (0.92–1.35)
No friends	—	0.73 (0.47–1.15)	0.71 (0.46–1.11)	0.71 (0.46–1.11)
Missed school	—	**2.26 (1.67**–**3.07)**	**2.20 (1.63**–**2.97)**	**2.19 (1.62**–**2.96)**
No parental monitoring	—	1.06 (0.80–1.42)	1.05 (0.77–1.42)	1.04 (0.76–1.41)
Drug use	—	**2.61 (1.89**–**3.60)**	**2.51 (1.82**–**3.47)**	**2.50 (1.80**–**3.45)**
Alcohol marketing				
Actors	—	—	1.26 (0.91–1.75)	1.27 (0.92–1.75)
Billboards	—	—	1.18 (0.84–1.65)	1.17 (0.84–1.64)
Provided free alcohol	—	—	**1.43 (1.07**–**1.90)**	**1.41 (1.06**–**1.87)**
Alcohol education				
Danger of alcohol	—	—	—	0.83 (0.63–1.10)
Refuse alcohol	—	—	—	1.11 (0.83–1.49)

Each model included all listed variables. Reference categories for each variable are not shown but were those not exposed to or who did not report bullying victimization, sadness, having no friends, missed school, no parental monitoring, drug use, alcohol marketing or alcohol education.
